# Electrical Broth Micro-Dilution for Rapid Antibiotic Resistance Testing

**DOI:** 10.1021/acssensors.2c02166

**Published:** 2023-02-23

**Authors:** Daniel Spencer, Yuetao Li, Yiling Zhu, J. Mark Sutton, Hywel Morgan

**Affiliations:** † School of Electronics and Computer Science, and Institute for Life Sciences, University of Southampton, Hants, Southampton SO17 1BJ, U.K.; ‡ Technology Development Group, Research and Evaluation, UK Health Security Agency (UKHSA), Porton, Salisbury SP4 0JG, U.K.; § Institute of Pharmaceutical Science, School of Cancer & Pharmaceutical Sciences, King’s College London, Franklin-Wilkins Building, 150 Stamford Street, London SE1 9NH, U.K.

**Keywords:** antimicrobial susceptibility test (AST), antimicrobial resistance, impedance sensing, broth microdilution. minimum inhibitory concentration (MIC)

## Abstract

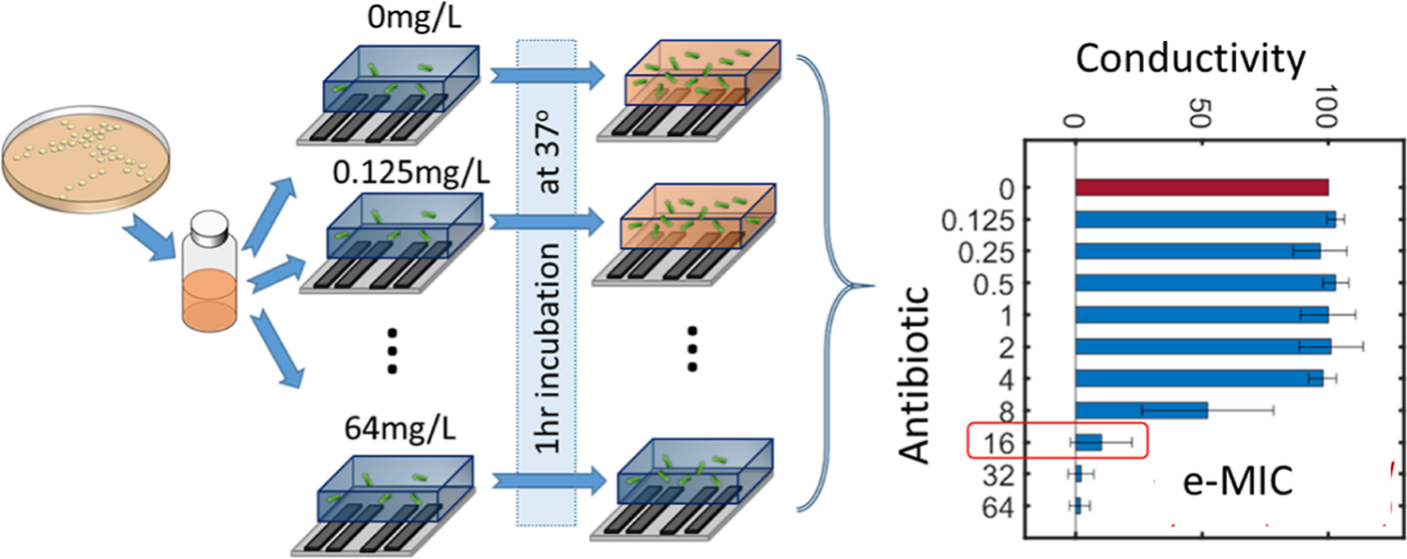

Rapid tests to assess the susceptibility of bacteria to antibiotics are required to inform antibiotic stewardship. We have developed a novel test, which measures changes in the impedance of a 100 nanoliter volume of bacterial suspension to determine an “electrical” minimum inhibitory concentration (eMIC). Two representative strains of *Klebsiella pneumoniae*, *Acinetobacter baumannii*, *Escherichia coli*, *Pseudomonas aeruginosa,* and *Staphylococcus aureus* were tested against a panel of frontline antibiotics with different modes of action (ciprofloxacin, doxycycline, colistin and imipenem, gentamicin, and ceftazidime). The eMIC measured at 1 h correlated strongly with a standard 24 h microbroth dilution MIC for all combinations of antibiotics and bacteria, allowing strains to be correctly assigned as sensitive or resistant measured in a fraction of the time.

## Introduction

In 2016, a report chaired by Jim O’Neill predicted that by 2050, anti-microbial resistance (AMR) will be the largest cause of death, killing 10 million worldwide annually.^1^ Three years later, in 2019, it has been estimated that 4.95 million people died from illnesses in which bacterial AMR played a part, with >1 million deaths as a direct result of AMR.^2^ Drug-resistant infections kill more people than HIV/AIDS (864,000 deaths) or malaria (643,000 deaths).

One of the recommendations from the O’Neill report was the need for faster and simpler diagnostics, including tests to determine the susceptibility profile of antibiotics.^3^ A rapid test would enable informed prescribing of therapy and reduce the ineffective treatment time window. The absence of a simple rapid diagnostic test drives the misuse of antibiotics, accelerating the emergence of antibiotic-resistant bacteria. Therefore, tests that can identify the most appropriate antibiotics are required to prevent inappropriate treatment and protect human health.

An antimicrobial susceptibility test (AST) determines the sensitivity of a bacterial pathogen to an antibiotic or panel of antibiotics. The classical AST is performed using either broth micro-dilution (BMD) or disk diffusion, where bacterial growth is measured in the presence of a range of concentrations of antibiotics. An AST is also used to determine the minimum inhibitory concentration (MIC) of an antibiotic. In BMD, this is defined as the lowest concentration of the antibiotic that inhibits the growth of an organism measured in vitro. This is measured by eye after an incubation time of typically 24 h. An AST is the “gold standard” but can take upward of 48 h to complete. Although widely used, they are time-consuming, principally because the number of bacteria does not reach the minimum detectable level, as defined by the standard protocols, for typically 24 h in rapidly growing bacteria.

Many rapid AMR tests have been reported in the literature, broadly categorized as either genotypic or phenotypic assays. Genotypic methods detect genes that confer resistance, while phenotypic assays monitor changes in the response or behavior of bacteria when exposed to antibiotics. Because the presence of a gene does not necessarily confer resistance, phenotypic tests are preferred to genotypic ones and are the most widely used in most settings. Several new tests are in development, and some have FDA approval and are commercially available,^4,5^ but the majority of tests still take many hours.

Microfluidic AMR tests have also been developed.^6^ Choi et al. described an optical system that could detect antibiotic susceptibility within 4 h.^7^ The system automatically counts bacteria by imaging morphological changes that occur after antibiotic exposure, such as division, swelling formation, and filamentary formation. Baltekin et al. designed a chip with 2000 parallel micrometer channels to trap and image single bacteria.^8^ The bacterium length was measured in the presence of an antibiotic and used to determine susceptibility within 30 min. Redox reporters such as resazurin have also been used to measure bacterial metabolism. Besant et al. designed an electrochemical device that captures bacteria in miniature wells and delivers a resistance profile after a 1 h incubation period.^9^ Droplets have been used as nL containers to trap single or small numbers of bacteria, with metabolism monitored by fluorescence.^10^ Other approaches use flow cytometry and metabolic probes to measure viability after exposure to antibiotics.^11^ However, all these methods require either the use of specific electrochemical or fluorescent labels, coupled with complex optical systems.

Electrical or electrochemical methods provide alternative methods of monitoring bacterial growth and behavior. They are relatively simple and do not require specific probes. Chotinantakul et al.^12^ described a method for detecting susceptibility to antibiotics using an electrochemical respiration assay following a 3 h incubation period. The growth of bacteria has also been measured directly using electrochemical impedance spectroscopy.^13,14^ Bacteria were cultured on antibiotic-seeded hydrogels and the susceptibility determined in a timeframe of around 2 h. Yang et al.^15^ demonstrated that bacteria trapped in a narrow channel can modify an electrical current flowing through the channel. As bacteria grow or die, the resistance changes, and the authors measured the response to ampicillin and nalidixic in a 2 h window. We have also shown that morphological changes in single bacteria induced by antibiotic exposure can be measured directly using single cell impedance spectroscopy.^16^

Rather than directly measuring the properties of the microorganisms, it is also possible to infer changes in properties from measurements of the suspending media. It has been known for nearly 100 years that as bacteria divide and grow, they produce a change in the electrical resistance of the suspending medium.^17^ The increase in biomass can also be directly measured by capacitance.^18,19^

**Figure 1:**
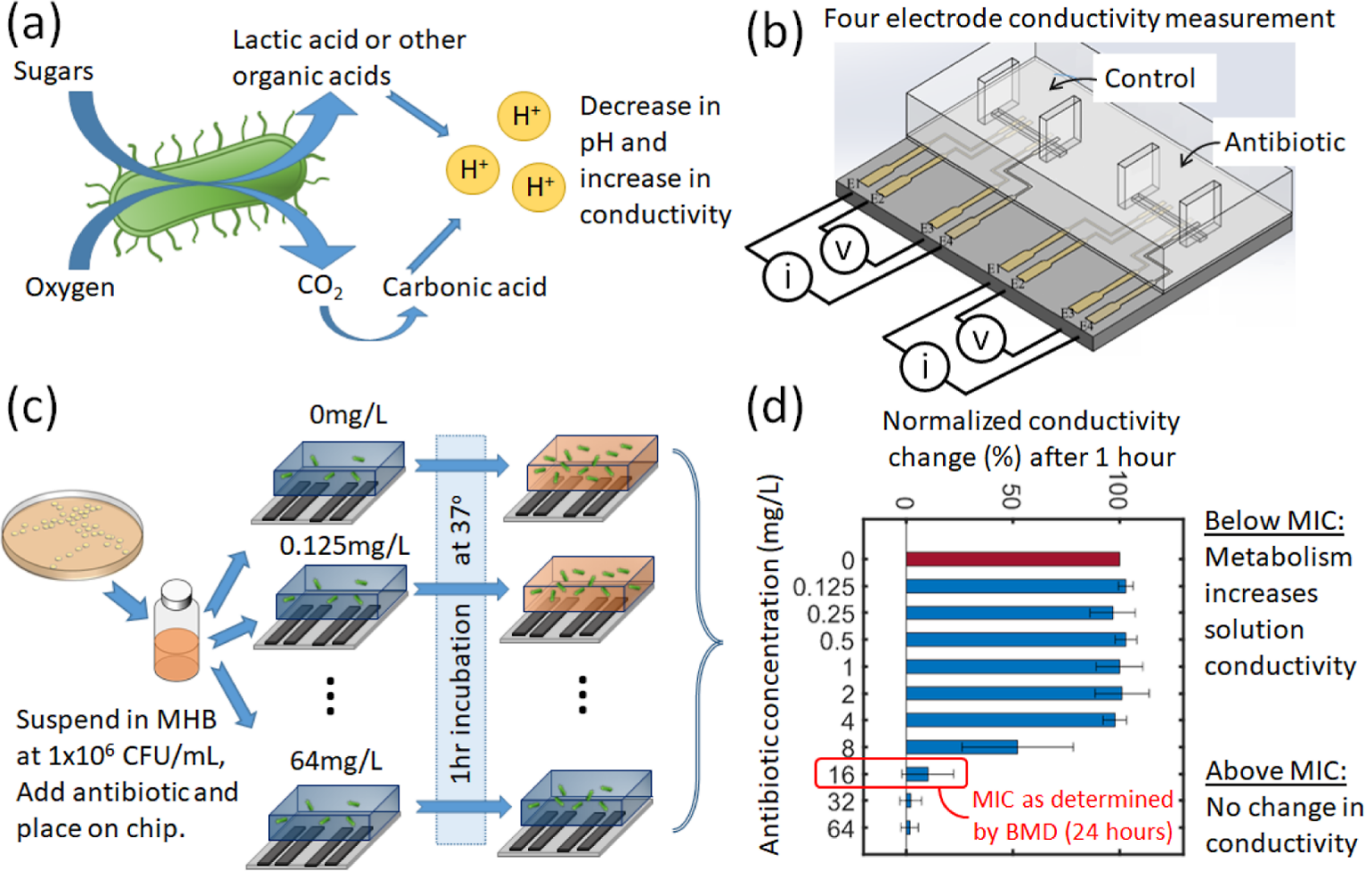
Principle of the rapid eMIC. (a) Actively dividing bacteria metabolize carbohydrates and other compounds, leading to a change in the pH of the conductivity of the suspending medium. This is measured with a four-electrode conductivity cell with a 100 nL measurement volume in a microfabricated glass chip, as shown in (b). (c) Graphical representation of the protocol: colonies from a plate are resuspended in Muller-Hinton broth to a concentration of 10^6^ CFU/mL and mixed with different concentrations of an antibiotic (diluted on a log_2_ scale from 0.125 to 64 mg/L) plus a control. (d) Typical result profile. At antibiotic concentrations below the MIC, the conductivity change after 1 h is similar for the antibiotic and control samples, reflecting active cell metabolism and growth. At higher concentrations, the antibiotic halts metabolism and there is no change in the suspension conductivity (0%).

Bacterial growth as determined from conductivity changes in the medium is due to the production of organic acids during metabolism, as illustrated in Figure 1a. Ur and Brown demonstrated concordance between measured growth and the electrical impedance of the suspending medium.^20,21^ The specific bacterial species and the presence of antibiotics all influenced the impedance. Cady et al. mixed bacterial samples with antibiotics and monitored the impedance over 20 h.^22,23^ They reported that an MIC could be determined by impedance at 5 h and that this was within a twofold difference to an MIC determined by BMD at 20 h. Richards et al. noted that conductivity changes dominate;^24^ Colvin and Sherris described a system to determine the MIC of antibiotics using impedance.^25^ After overnight incubation, there was 93% correlation with visual readings. When the time window was reduced to 6 h, the correlation dropped to only 34%, but this was improved by increasing the inoculum concentration.

Miniature interdigitated electrode arrays have been used to improve the sensitivity and simultaneously capture and concentrate bacteria from solution using electrophoresis or dielectrophoresis.^26^ Sengupta et al. showed that the concentration of bacteria could be measured by capacitance and that growth in the presence of antibiotics could be used to determine resistance and an MIC.^27^ They showed different changes in capacitance over time and attributed these to differences in the mode of action of the antibiotic, either bacteriostatic or bactericidal.

Safavieh et al. developed a simple AMR test by measuring the impedance of bacteria captured using antibodies onto an interdigitated electrode array. They demonstrated that the medium resistance of an *E. coli* suspension or a methicillin-resistant *Staphylococcus aureus* (MRSA) suspension reduced by >50% within 1 h, when the initial bacterial concentration was 10^6^ CFU/mL.^28^ Mixing antibiotics with the bacterial samples significantly slowed the resistance change in susceptible bacteria. Their device could detect antibiotic susceptibility of *E. coli* and *S. aureus* based on an end-point impedance after 90 min. Scherer et al. developed a multiplexer circuit to measure many nanoliter chambers in parallel.^29^ In contrast to an increasing medium conductivity during growth, Swami et al. used a low-conductivity suspending medium to measure the release of ions following cell death on a miniature interdigitated electrode array.^30^

In this paper, we describe a simple test that provides an “electrical” MIC (eMIC) by measuring the change in the electrical impedance of a bacterial suspension after a short 1 h exposure to a range of antibiotic concentrations. The protocol was designed to mirror the classical BMD used in diagnostic microbiology laboratories and described by standard ISO 20776-1:2019.^31^ During growth, bacteria metabolize sugars to lactic acid or other organic acids and other compounds that decrease the pH and increase the conductivity of the suspending media (Figure 1a). However, for slowly dividing or non-viable bacteria that have been exposed to antibiotics, this change in conductivity is small or non-existent. Both the resistance and capacitance of a suspension of bacteria can be influenced by many factors other than the antibiotics, including temperature. In order to obviate these issues, we developed a simple difference measurement technique that cancels out any non-biological trends. Two samples of identical bacteria are measured together. One is exposed to antibiotics, while the other (control) sample is not. The difference between the two impedance signals after 1 h of incubation indicates the degree of susceptibility or resistance.

## Results and Discussion

The conductivity of a suspension of bacteria in standard growth medium was measured with a miniature sample cell, shown conceptually in Figure 1b. The “sensor chip” consists of sets of platinum micro-electrodes fabricated onto a glass substrate. Each sample well contains one set of four electrodes that measures the electric impedance of the sample at low AC frequencies. Two outer electrodes inject an AC current, while a pair of inner electrodes is used to measure the voltage drop. Two sensors are used simultaneously, one containing the antibiotic exposed sample and the other the control (non-exposed) sample. The sensing chip is fabricated so that a narrow channel runs over the electrodes (see Methods). The total volume of the channel is 2 μL, but the sample volume above the sensing electrodes is approximately 100 nL. Samples of bacteria are injected into the device with a pipette, and the entry and exit ports are covered with a thin layer of mineral oil to prevent evaporation. The impedance is measured at time zero and again after 1 h. Chips can be washed and reused. For further details, see Methods.

In a typical test, a colony of bacteria from an overnight culture is resuspended in MH1 broth to an optical density (OD) equivalent to approximately 10^6^ CFU/mL. Aliquots are mixed with specific antibiotics at different concentrations including a control sample with no antibiotics, and samples introduced into the chip. The chips are incubated for 1 h at 37 °C on a heated plate, which is part of the reader (see Methods). This protocol is shown in Figure 1c. The change in impedance of the antibiotic-exposed samples is measured (100 mV at 100 Hz) and referenced against an identical control sample with no antibiotics. The magnitude of the impedance at the start of the test ( and at the end ( was normalized to the control (0 mg/L), according to the following equation (the phase angle is always close to zero).
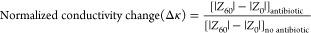
1

Bacteria that are resistant to antibiotics continue to grow and metabolize, and the change in impedance in both control and antibiotic exposed is almost the same, so that Δκ is close to 100%. Any change in growth due to the presence of antibiotic reduces Δκ, and for fully susceptible bacteria, this is close to 0%.

Figure 1d shows an example dataset for *Klebsiella pneumoniae* strain NCTC 13368 exposed to ceftazidime at a range of different concentrations. The *x*-axis shows the normalized conductivity change (eq 1 ) and the *y*-axis is the log_2_ fold concentration range of antibiotics, as per a standard BMD. The error bars show the standard deviation for *n* = 3 biological repeats. By definition, the first bar (antibiotic concentration = 0 mg/mL) is set to 100%. The graph shows that for low antibiotic concentrations, the change in conductivity is similar to the control. At higher antibiotic concentrations, the change after 60 min is much lower. In other words, the antibiotic exposed cells do not grow (or die), while the control cells continue to grow leading to a large (differential) change in the conductivity of the suspending medium. Overlaid on the image is the MIC determined by a classical broth microdilution (after 24 h) for the same sample (16 mg/L) demonstrating excellent concordance with the electrical MIC (or eMIC). Having demonstrated the principle of the system, the *Klebsiella* response was measured for six different antibiotics representing a wide range of modes of action. The antibiotics were *ceftazidime*, *colistin*, *ciprofloxacin*, *gentamicin*, *imipenem,* and *doxycycline*, and their modes of action are summarized in Table 1.

**Table 1:** Summary of Mode of Action for Different Antibiotics Used

ceftazidime	inhibition of cell wall synthesis by binding to penicillin-binding proteins
ciprofloxacin	fluoroquinolone that acts on topoisomerases (DNA gyrase) preventing supercoiling of the DNA and DNA replication
colistin	targets cell membrane binding to LPS and phospholipids displacing divalent cations leading to disruption of the outer cell membrane and death
doxycycline	bacteriostatic (rather than bactericidal). Stops bacterial growth by binding to the 30S ribosome. Lipophilic so easily crosses multiple membranes
gentamicin	inhibits protein synthesis by binding 30S ribosomes
imipenem	inhibition of cell wall synthesis by binding to penicillin-binding proteins

**Figure 2:**
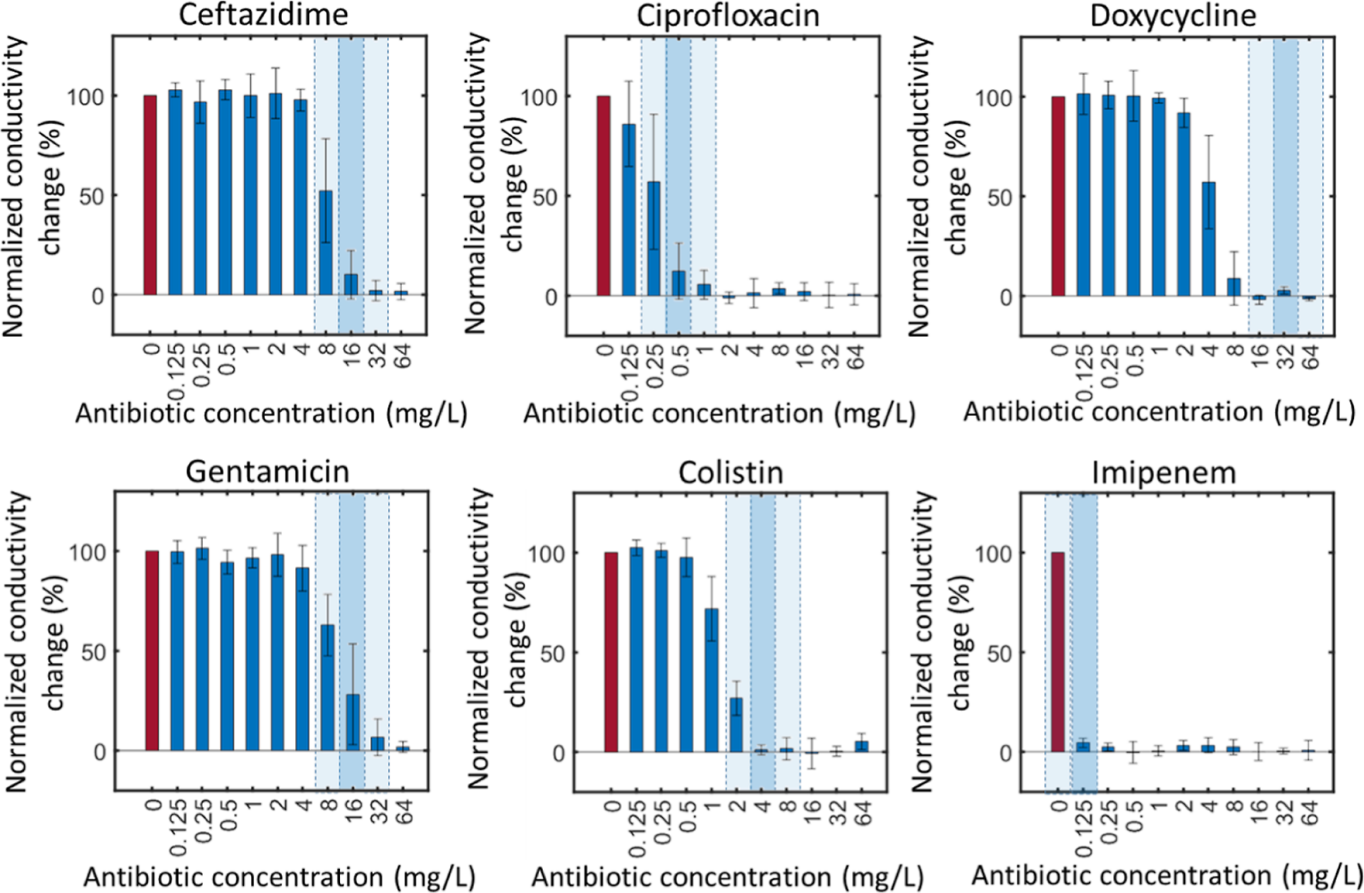
Bar chart showing the relative growth of the *Klebsiella pneumonia* strain 13368 after a 1 h exposure to six different antibiotics at 10 different concentrations (and a control of 0 mg/L). The *y*-axis represents the normalized conductivity change at 60 min, which is 100% for the unexposed sample and is shown as the red bar for clarity. The height of the bar is the mean for *n* = 3 biological repeats, and the error bar shows the standard deviation. The MIC for each sample as determined by BMD (after 24 h) is shown by the shaded dark-blue regions. The light blue regions either side highlight the ±1 dilution band, corresponding to the accepted tolerance of the BMD.

Figure 2 shows the conductivity data for *K. pneumoniae*; NCTC 13368 exposed for 1 h to each of these different antibiotics. The height of the bars indicates the response of the bacteria as measured by changes in conductivity, normalized to 100% according to eq 1. The bars are the mean of three repeats with one S.D. Also shown is the MIC for each sample as determined by BMD (after 24 h); shaded dark-blue regions in the figure. The light blue regions either side highlight the ±1 dilution band, corresponding to the accepted tolerance of the broth MIC method, where a twofold difference in MIC is not generally regarded as significant (see ISO 20776-1:2019^31^). The conductivity change across the dilution range follows a sigmoidal shape, where the drop in conductivity mirrors observations made with classical broth microdilution. For all antibiotics, the conductivity change starts to drop at sub-MIC concentration and falls below 50% at the MIC; little or no change in conductivity is observed at antibiotic concentrations above the MIC. It is noteworthy that despite the wide range of different antibiotics used, the general method of measuring the impedance change provides an eMIC that is consistent (within one dilution) with the BMD. Of note is the response to imipenem, a carbapenem that inhibits growth at even the lowest antibiotic concentration used in this study (0.125 mg/mL, corresponding to the broth MIC), where the relative conductivity change during 1 h is almost zero. All six antibiotics lead to observable differences in suspending medium conductivity relative to the control, but there does not appear to be anything to link the changes to the mode of action of the antibiotic apart from doxycycline. This is a bacteriostatic antibiotic, which does not kill the cells but inhibits division. Even in this case, there is a significant change in the impedance after the 1 h incubation window. The observed changes in impedance are a function of the metabolic state and growth of the bacteria, so they should be similar for most bacteria irrespective of the resistance mechanism. The only exception might be resistance mechanisms which are switched on more slowly by bacteria, such as vanA expression in Enterococcus.

**Figure 3:**
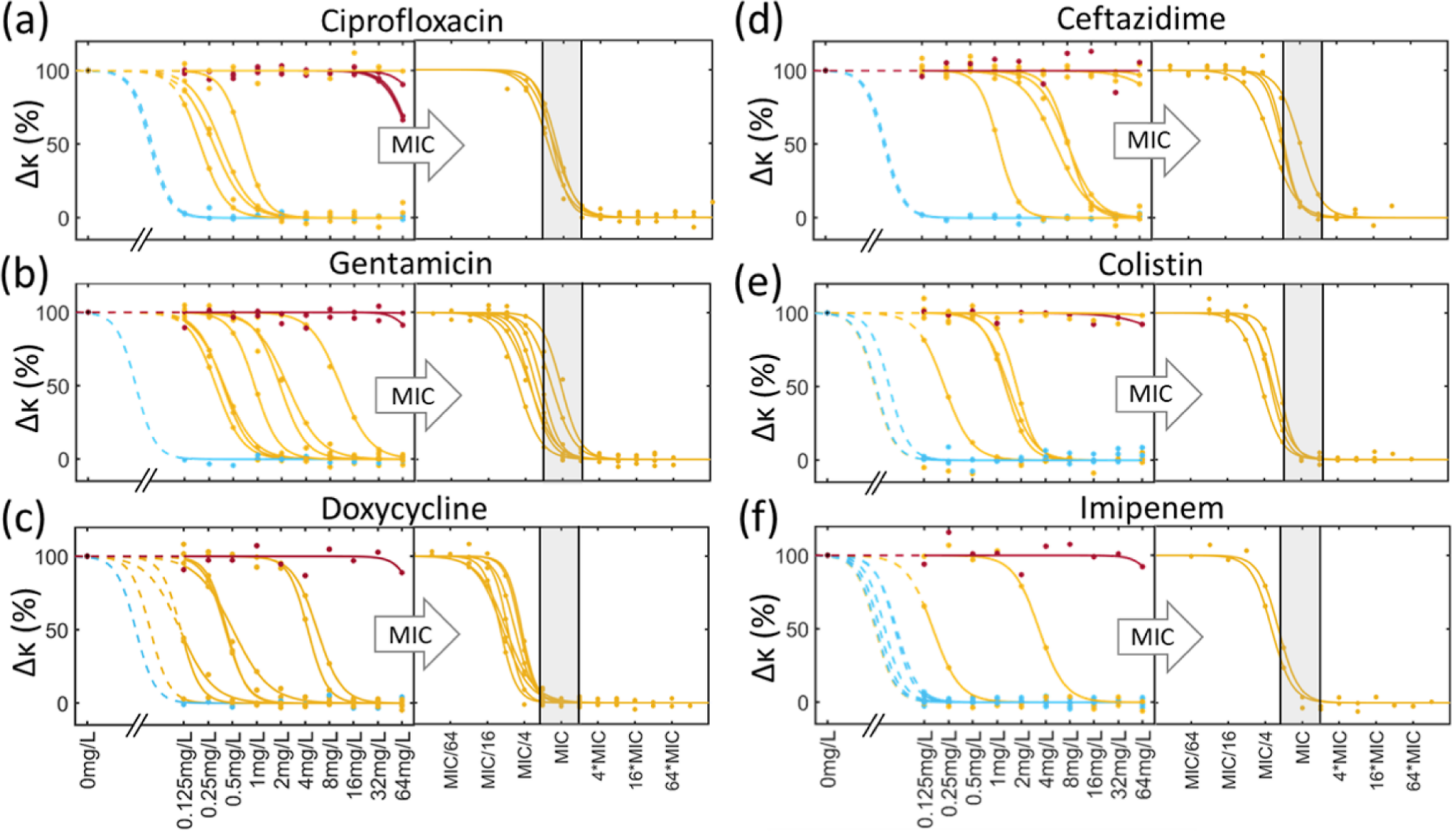
Normalized impedance change for 10 different bacteria (5 species, 2 strains of each), exposed to six different antibiotics. Each line is for a different organism (not labeled for clarity, see Supporting Information for the complete data set). The lines are color coded as per the MIC determined by a standard broth microdilution (BMD), where blue is highly sensitive ≤0.125 mg/L and red is highly resistant >64 and 0.125 mg/L < MIC ≤64 mg/L in yellow. For each of (a–f), the normalized conductivity change is plotted against the absolute antibiotic concentration (lhs) and also against the MIC (rhs) determined from the BMD.

To determine the equivalence between our rapid readout (electrical metabolism) and classical methods, a range of different bacterial species was measured consisting of five different species (two strains of each) using the same six antibiotics. The panel was *K. pneumoniae* (NCTC 13368 and M6), *E. coli* (NCTC 12923 and LEC001), *S. aureus* (EMRSA-15 and ATCC 9144), *A. baumannii* (AYE and ATCC 17978), and *P. aeruginosa* (PAO1 and NCTC 13437). The original data (impedance before normalization) for all 10 bacteria and 6 antibiotics are in the Supporting Information, Figure S1, where the *y*-axis is the absolute change in impedance (not normalized). This entire data set can be consolidated into a set of six plots, as shown in Figure 3 where the normalized electrical impedance change is plotted against antibiotic concentrations for all different microorganisms. For each of the six antibiotics (a–f). There are two plots. For each plot, the left-hand side panel is the normalized electrical impedance versus absolute antibiotic concentrations. For clarity of presentation, these lines have not been labeled with the name of organism (refer to Supporting Information for individual plots) but are color coded as per the MIC determined from a standard broth microdilution.^32^ The blue color indicates highly sensitive MIC ≤0.125 mg/L, red highly resistant MIC > 64 mg/L, and yellow intermediate 0.125 mg/L < MIC ≤64 mg/L. The rhs panel shows the data scaled to the MIC for the case where this is explicit (i.e., the yellow lines), not for the blue or red lines as the MIC for these very sensitive or resistant strains are not exact (i.e., ≤ minimum or > maximum test concentration). The graph shows that the data collapse onto a single set of sigmoidal curves, demonstrating the effectiveness of the rapid e-MIC method for strains with intermediate MICs.

For ciprofloxacin, doxycycline, colistin, and imipenem, the curves overlap within a very tight band, whereas for gentamicin and ceftazidime, the band is slightly wider. The reasons for this are not clear, but one possible explanation could be the presence or relative timing of expression of different resistance mechanisms, which affect the conductivity measurement for specific isolates. For doxycycline, a bacteriostatic antibiotic, the mean change occurs at around MIC/4, while at the MIC, there is close to 0% growth. The MIC is an end-point measurement after typically 24 h and considers any growth up to that time point. Therefore, our observation of partial growth (at sub-MIC concentrations) is not unexpected. For ciprofloxacin at the MIC, the conductivity change is in the range of 30–10%, falling to 0% at 2× MIC. This difference may reflect different modes of action of the antibiotics and could be compensated for in any future clinical test. Despite this, the data demonstrate the utility of a fast electrical-MIC test, where setting the threshold for conductivity to, e.g., 10% would correctly identify the MIC within +/– twofold dilution.

**Figure 4:**
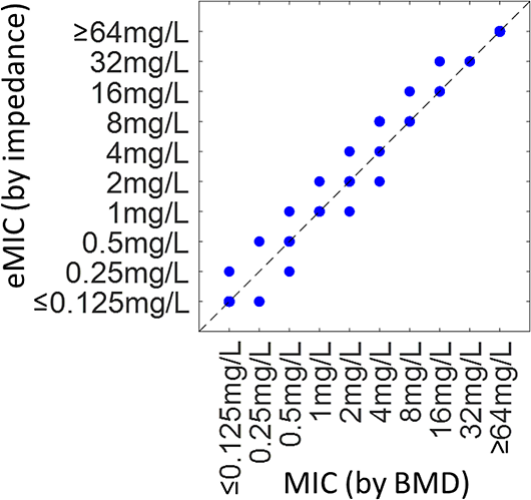
Correlation between the classical MIC determined by BMD at 24 h and the eMIC determined after 1 h of incubation by impedance. The eMIC is defined (from Figure 3) as the antibiotic concentration for which the normalized impedance (Δκ) falls below a threshold (see text for details). Note that many data points overlap.

From the data in Figure 3, it is not possible to determine an exact value for the electrical MIC; this requires defining a threshold in the impedance data (Δκ). In classical broth microdilution, the threshold is defined as the point at which growth is no longer observable by eye. To set a similar threshold, we calculated the average value of Δκ for the set of yellow impedance curves using the MIC determined by BMD—see Figure S2 for further details. Figure 3 shows that impedance sensitivity depends on the antibiotic, i.e., the impedance reduces earlier for some antibiotics compared with others. Therefore, the same calculation was done separately for each antibiotic. We then defined the eMIC as the lowest antibiotic concentration at which Δκ is below this threshold (see Figure S2). Finally, the correlation between the eMIC and MIC is plotted in Figure 4 for all antibiotic/strain combinations. The figure shows that the eMIC matches the classical MIC within one dilution for all cases. This is termed essential agreement by ISO 20776-1:2019. Note that these data have been collected for a very small panel of antibiotics and strains, but they demonstrate the principle of the technique. The threshold for other organisms/antibiotic combinations would need to be determined explicitly by measuring an extensive panel and is likely to be different for each combination.

The test is designed to integrate into a conventional clinical workflow, typical of any microbiology diagnostic laboratory in a large hospital. A patient sample such as urine is first pre-processed with the causative organisms grown on agar plates to produce pure cultures. Typically, this may be a low electrolyte agar, for example, a CLED plate (cystine–lactose–electrolyte-deficient agar) that is a non-inhibitory growth medium for the isolation and differentiation of urinary *Enterobacteriaceae* and *Pseudomonas*. After 24 h, colonies are then re-streaked onto conventional agar plates for disk diffusion experiments or alternatively, suspended in media for a BMD assay, which takes another 18–24 h. For blood, a 10 mL blood bottle (containing growth medium) is kept at 37 °C until bacterial growth is detected (from a pH change due to CO_2_), which can take many days. A classical AST is performed when sufficient organisms are present. Because our assay requires fewer total organisms, it does not require the full 24 h incubation on a purity plate and could be integrated into a standard clinical workflow. For example, a rapid AST would be performed on organisms taken from a CLED plate after a short growth period (a few hours), thus significantly shortening the time of the AST, potentially from nearly 2 days to a few hours (within a clinical shift).

## Conclusions

This paper has demonstrated a very simple, inexpensive, and rapid method of determining the MIC of bacteria by measuring the change in the electrical conductivity of the suspending medium containing a few hundred organisms. The test was evaluated with a panel of 10 different bacterial strains and 6 antibiotics, demonstrating excellent equivalence with the classical broth microdilution assay. The measurement chamber volumes are small (100 nL) and use standard concentrations of bacteria (10^6^ cfu/mL), making the technology suitable for applications where few bacteria are available, for example, susceptibility testing of bacteria in blood.^33^ The electrical measurements can be easily scaled and multiplexed to monitor a large number of chambers simultaneously, which is ideal for the high-throughput requirements for clinical microbiology laboratories. The current system is designed to fit into a standard clinical workflow, where colonies are picked from overnight purity plates. The development of a rapid test that is direct from the sample would require additional methods of extracting and purifying bacteria prior to incubation in media.

## Methods

### Chip Sensor Design

The measurement chip shown in Figure 1b has two chambers side by side, one for the control (no antibiotic) and one for the exposed (with antibiotic) bacterial sample. The base of the chip comprises a glass substrate, onto which pairs of platinum microelectrodes are fabricated by photolithography. Each electrode is 200 μm wide; the distance between the measurement electrode pair is 1.2 mm, and the outer (drive) electrode pair center-to-center spacing is 2 mm. A measurement chamber is made from PMMA bonded to the glass substrate using double-sided medical grade adhesive tape (3 M). The PMMA chamber is designed to be filled with approximately 2 μL of the sample, but the measurement chamber volume (covering the electrodes) is only approximately 200 nL (125 μm deep, 4 mm long, and 400 μm wide). The detection volume (above the two sensing electrodes) is approximately half of the culture chamber volume (around 100 nL). Bacteria are loaded into the chamber at a concentration of approximately 10^6^ cfu/mL in MH1 media using a pipette. A minimum of two samples is required, one without antibiotics (control) and one (or more) with fixed concentrations. After filling the inlet and outlet chambers, they are covered with a thin layer of mineral oil to prevent evaporation. Each chip measures a single antibiotic at a single concentration; multiple chips are connected in parallel, and signals are processed through a multiplexer. A simple electronic circuit provides a constant current drive signal of 100 mV at 10 Hz to the outer electrode pair, with the impedance magnitude determined from the voltage measured across the inner electrode pair. The real part of the impedance is the sample conductivity (phase angle is zero). The measurement chips sit on a heated pad (Kapton Polyimide Flexible Heater, Omega USA) to maintain a temperature of 37 °C via a PID controller (Red Lion PXU30020 USA). A type K thermocouple (TENMA) was placed directly on the chip (with thermal paste) and used to control the PID. A photograph of the final assembly is shown in Supporting Information, Figure S3.

### Chip Calibration

The sensor performance was evaluated using conductivity calibration solutions. The impedance magnitude vs frequency is shown in Supporting Information Figure S4, demonstrating that the signal is independent of the frequency below 100 kHz and that the measured signal is dominated by the real part of the impedance. The accuracy of the system was evaluated by plotting the difference in the impedance signal compared to a commercial conductivity meter (RS PRO 123-8777) with calibration solutions (Hannah Instruments). For values of conductivity around 0.5 S/m, the error was less than 1% (see Supporting Information). Note that for the measurement of bacteria, only the relative change in conductivity with time is required (not the absolute value) so that the accuracy is less important.

### Broth Micro Dilution

*K. pneumoniae* (NCTC 13368 and M6), *E. coli* (NCTC 12923 and LEC001), *S. aureus* (EMRSA-15 and ATCC 9144), *A. baumannii* (AYE and ATCC 17978), and *Pseudomonas aeruginosa* (PAO1 and NCTC 13437) (as described previously^34^) were used for resistance/susceptibility testing using a modified version of the CLSI standard method, with MH1 media replacing MH2 to facilitate comparison with the conductance measurements. The strains were cultured in a shaking incubator at 200 rpm overnight at 37 °C in 3 mL MH1 broth. The OD of overnight culture was determined at 600 nm. In a 96-well plate, 200 μL of bacterial suspension in MH1 broth, with a final OD equivalent to 5 × 10^5^ cfu/mL, were incubated with antibiotics at 64 μg/mL to 0 μg/mL. The endpoint OD_600_ of each well was recorded after the 96-well plate was incubated at 37 °C for >20 h.

## Supporting Information Available

Percentage impedance change for five different species of bacteria; method used to determine the electrical MIC; photograph of the final experimental setup showing a chip with six sensors; and plot of the magnitude of impedance and phase angle for MH1 medium as a function of frequency (PDF)

## Notes

The data that support the findings of this study are openly available in University of Southampton repository at https://doi.org/10.5258/SOTON/D2538.
