# Supplementary material for: Electrical Broth Micro-Dilution for Rapid Antibiotic Resistance Testing
Source: ACS Sens. 2023 Feb 23;8(3):1101–8. doi: 10.1021/acssensors.2c02166 (PMC10043929; doi:10.1021/acssensors.2c02166)

Supporting Information

Electrical broth micro-dilution for rapid antibiotic resistance testing

Daniel Spencer[a], Yuetao Li[a], Yiling Zhu[b], J. Mark Sutton[b,c], Hywel Morgan[a,*]

[a] School of Electronics and Computer Science, and Institute for Life Sciences, University of Southampton, Hants, SO17 1BJ UK

[b] Technology Development Group, Research and Evaluation, UK Health Security Agency (UKHSA), Porton, Salisbury, SP4 0JG UK

[c]Institute of Pharmaceutical Science, School of Cancer & Pharmaceutical Sciences, King's College London, Franklin-Wilkins Building, 150 Stamford Street, London, SE1 9NH, United Kingdom

Figure S1 A-E. The percentage impedance change determined from as $\frac{[|Z_{t=60\min}| - |Z_{t=0min}|]}{|Z_{t=0min}|}$ (%) for five different species of bacteria (2 strains of each – see labels), exposed to 6 antibiotics with 10 antibiotic concentrations (and 0mg/L). The MIC determined by a standard broth microdilution is indicated as circles on the x-axis (colour coded).

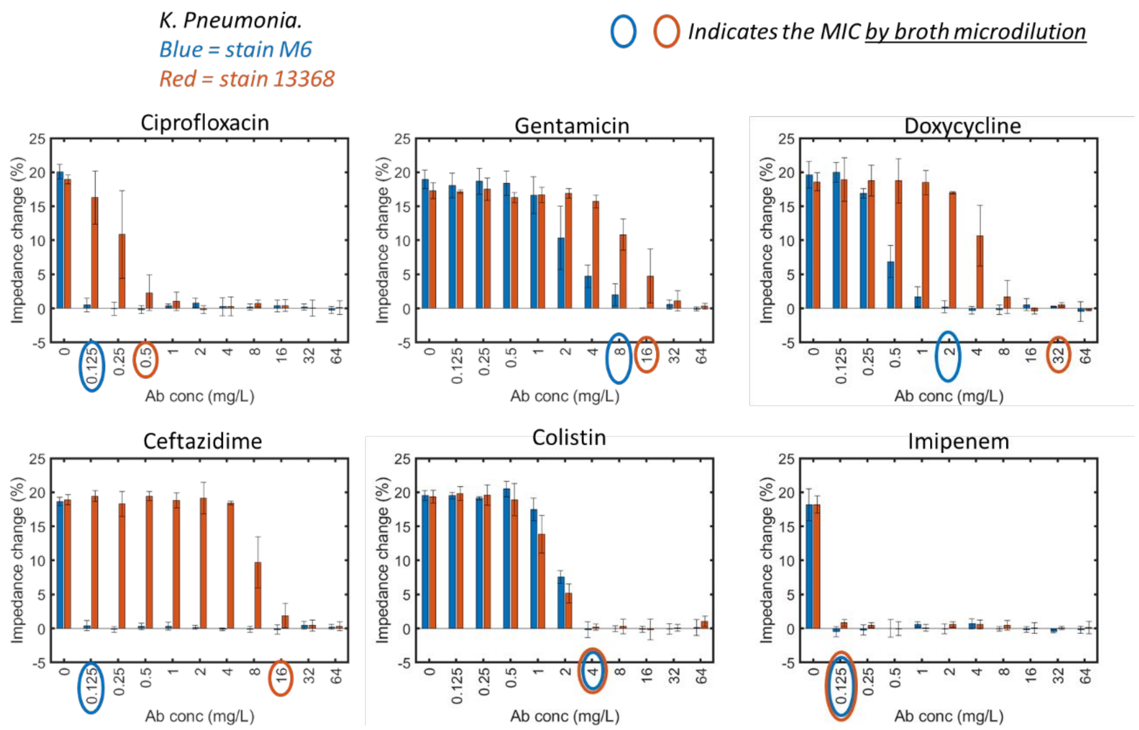

E. Coli.

Blue = stain LEC-001

Red = stain 12923

Indicates the MIC by broth microdilution

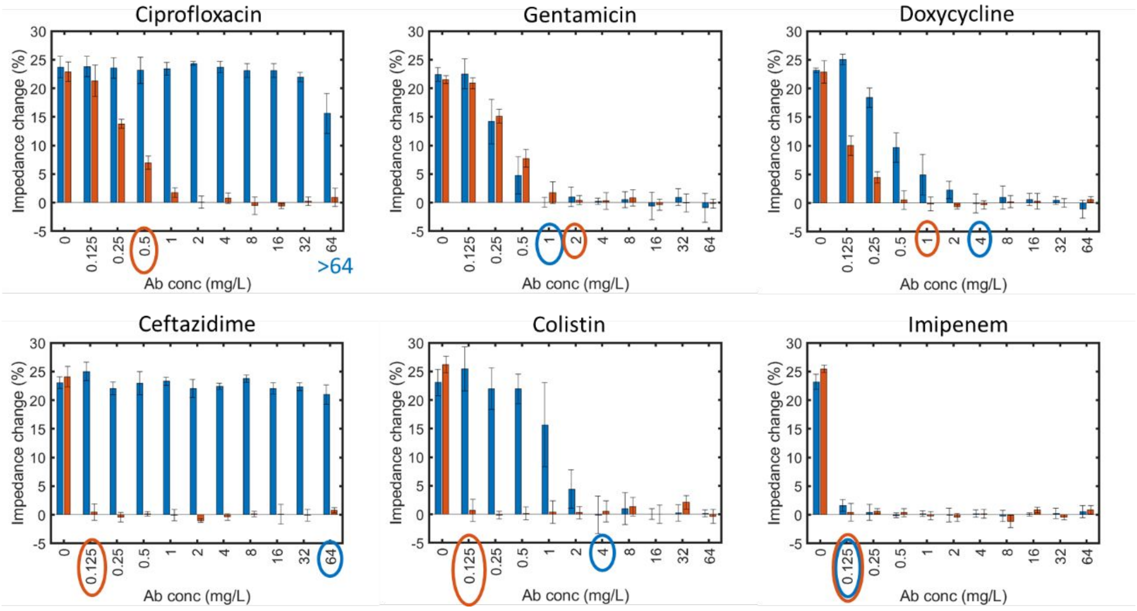

S. Aureus

Blue = stain EMRSA-15

Red = stain 9144

Indicates the MIC by broth microdilution

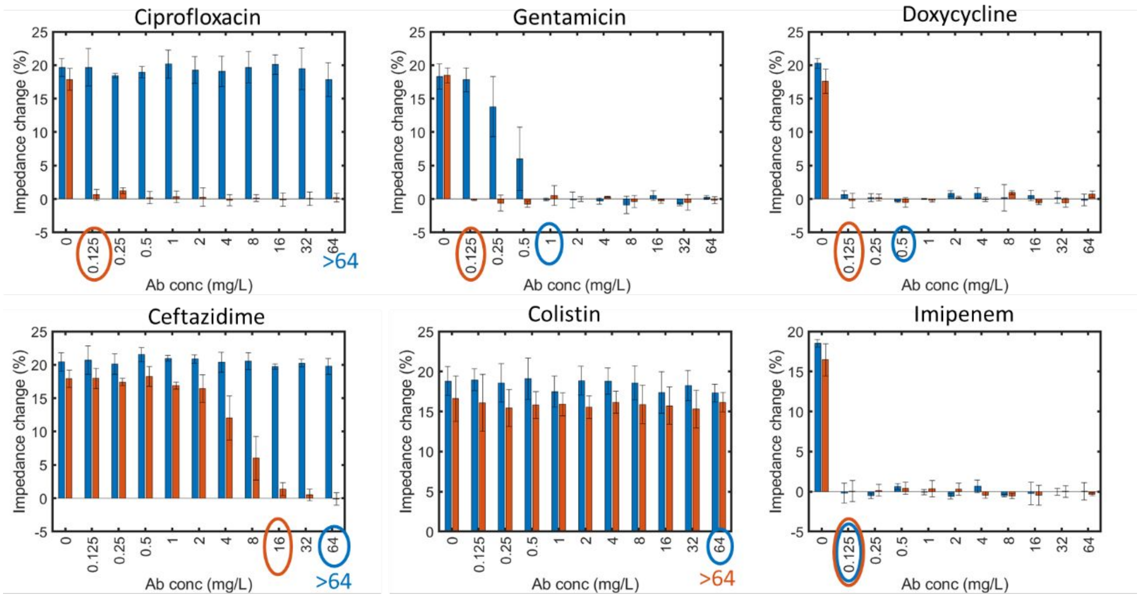

A. baumannii
Blue = stain AYE
Red = stain 17978

Indicates the MIC *by broth microdilution*

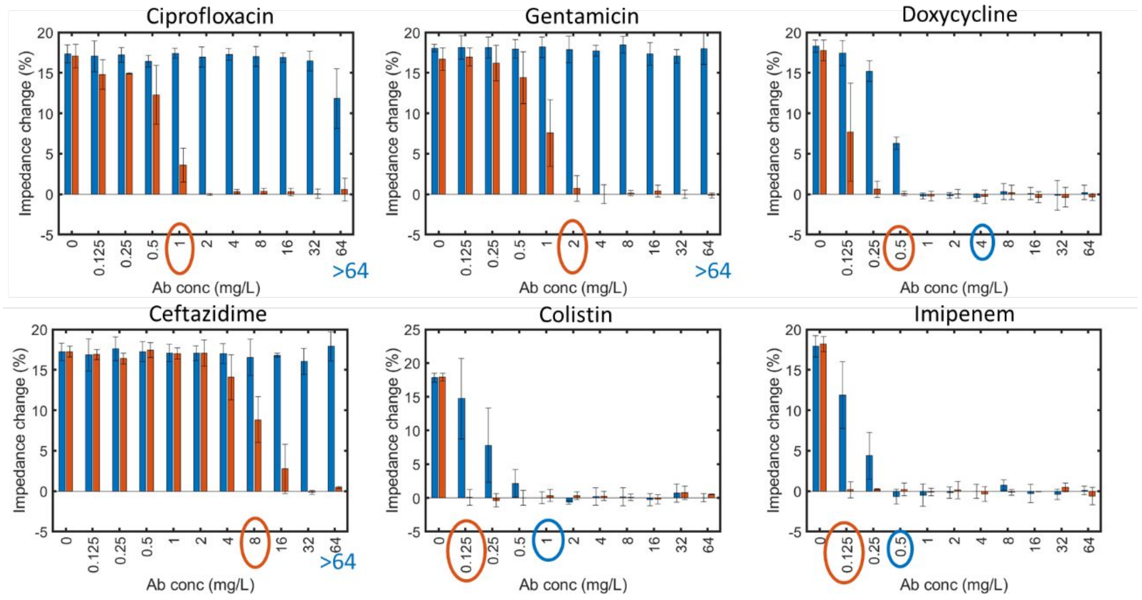

Pseudomonas
Blue = stain PA01
Red = stain 13437

Indicates the MIC *by broth microdilution*

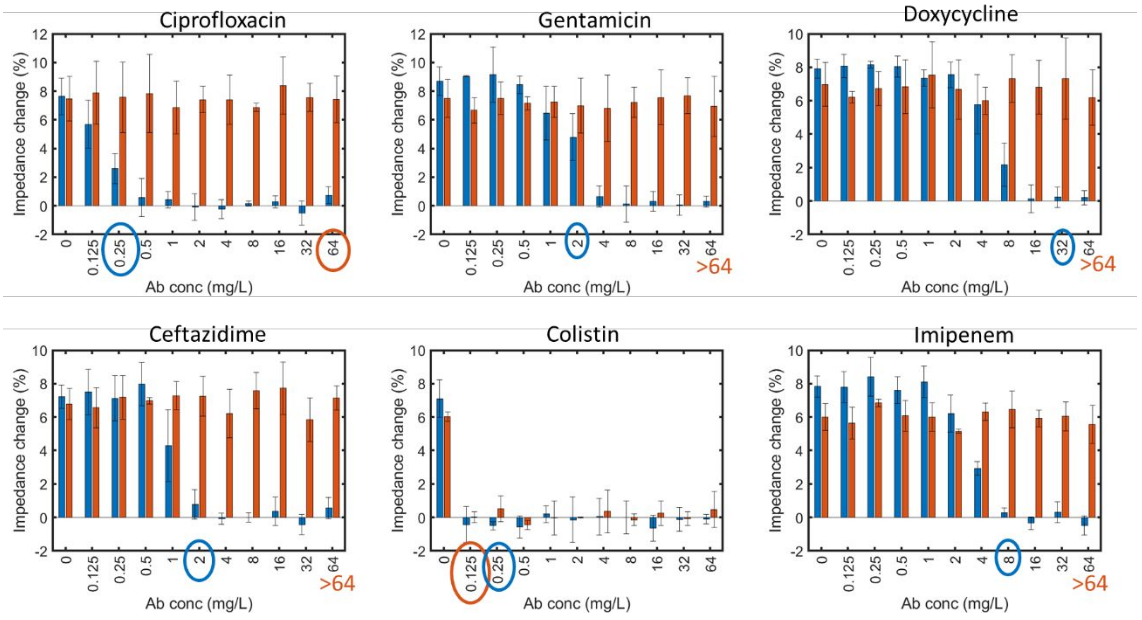

Figure S2. Method used to determine the electrical MIC (eMIC). The mean of the Δκ was calculated at the classical MIC value (a). This is shown by the star symbol. The lowest concentration of antibiotic with Δκ below the threshold is defined as the eMIC – see symbols on (b)

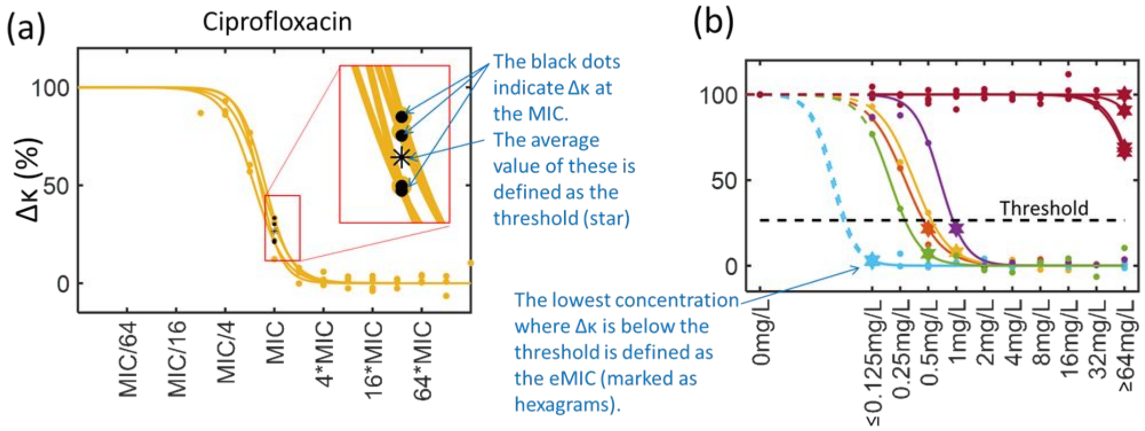

Figure S3. Photograph of the final experimental setup showing a chip with 6 sensors (1 sensor per sample), mounted on top of a resistive heater pad controlled by a thermocouple.

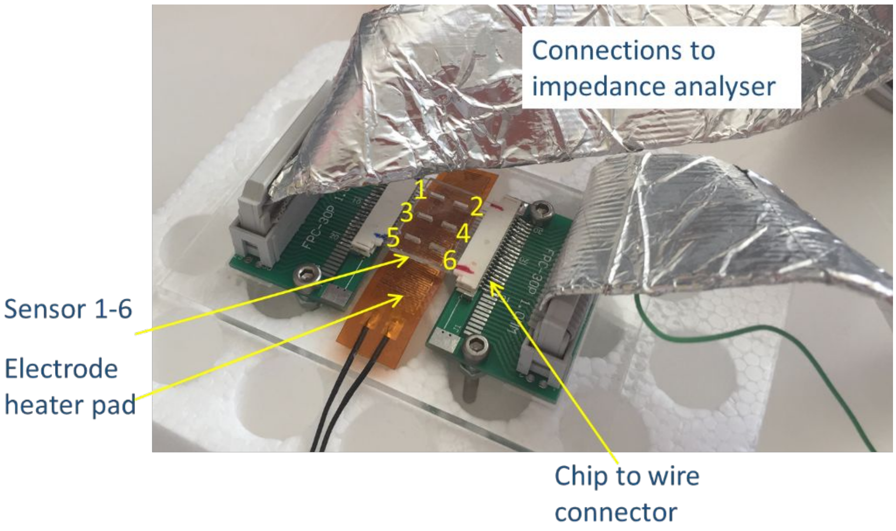

Figure S4. Plot of the magnitude of impedance and phase angle for MH1 medium as a function of frequency demonstrating the impedance magnitude corresponds to the conductivity at low frequencies. Also shown is a demonstration of the error when measuring commercial calibration solutions

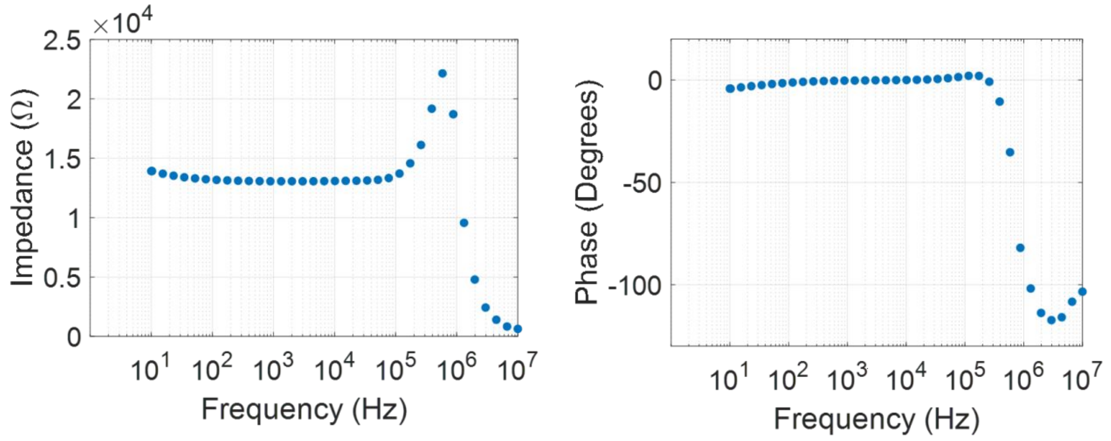

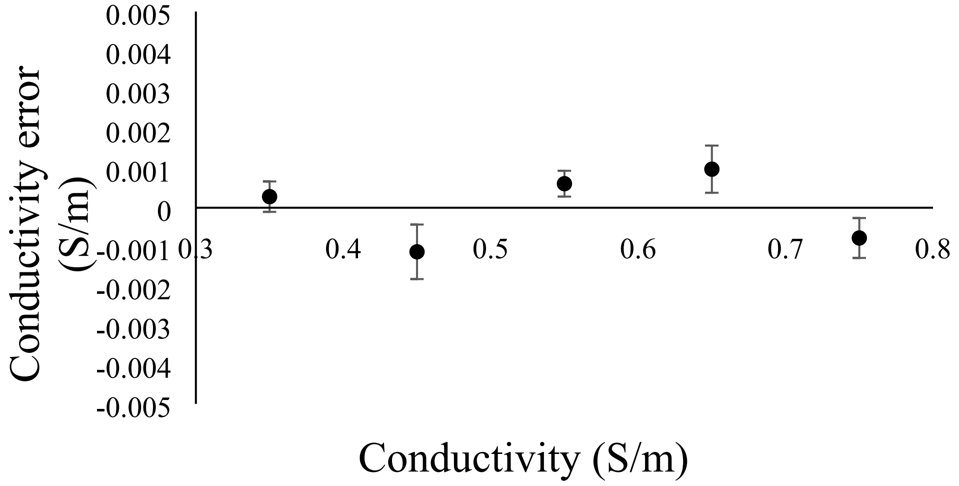

Supplement: Supplementary file 1 — se2c02166_si_001.pdf [file se2c02166_si_001.pdf]
